# Diffusion tensor imaging for detecting biomarkers of idiopathic epilepsy in dogs

**DOI:** 10.3389/fvets.2024.1480860

**Published:** 2025-01-07

**Authors:** Grace T. Kadler, Alex zur Linden, Luis Gaitero, Fiona M. K. James

**Affiliations:** ^1^Department of Biomedical Sciences, Ontario Veterinary College, University of Guelph, Guelph, ON, Canada; ^2^Department of Clinical Studies, Ontario Veterinary College, University of Guelph, Guelph, ON, Canada

**Keywords:** idiopathic epilepsy (IE), dogs, neuroimaging, biomarker, microstructure, diffusion tensor imaging (DTI), drug resistant epilepsy (DRE), fractional anisotropy (FA)

## Abstract

Idiopathic epilepsy (IE) is the most common neurological disease in dogs. Approximately 1/3 of dogs with IE are resistant to anti-seizure medications (ASMs). Because the diagnosis of IE is largely based on the exclusion of other diseases, it would be beneficial to indicate an IE biomarker to better understand, diagnose, and treat this disease. Diffusion tensor imaging (DTI), a magnetic resonance imaging (MRI) sequence, is used in human medicine to detect microstructural biomarkers of epilepsy. Based on the translational model between people and dogs, the use of DTI should be investigated in a veterinary context to determine if it is a viable resource for detecting microstructural white matter abnormalities in the brains of dogs with IE. As well, to determine if there are differences in white matter microstructure between dogs who are responsive to ASMs and dogs who are resistant to ASMs. Using DTI to better understand neurostructural abnormalities associated with IE and ASM resistance might help refine diagnostic approaches and treatment processes in veterinary medicine.

## 1 Introduction

Epilepsy is one of the most common neurological diseases in dogs (1). It reduces quality of life and shortens a dog’s lifespan (1–5). In veterinary medicine, idiopathic epilepsy (IE) is diagnosed indirectly on criteria that excludes evidence of alternative diseases (6). Therefore, finding biomarkers that could further narrow down the diagnosis of IE would be clinically useful. One option, diffusion tensor imaging (DTI), is used in human medicine to help researchers and doctors better understand brain connectivity and diseases that affect white matter such as epilepsy in people (7–21). Epilepsy arises naturally in both people and dogs; strong arguments exist as to the similarity of the disease between the two species (22–26). Thus, DTI may offer an opportunity to detect microscopic neurostructural abnormalities in dogs that support the diagnosis of IE and improve treatment planning. Furthermore, DTI-detected abnormalities may partially explain why some dogs are responsive to anti-seizure medications (ASMs) while others are resistant as differences have been reported in a couple of clinics trials on people (14, 19). The use of DTI for investigating white matter abnormalities in dogs with IE is in the early stages of exploration (27). In this narrative review, background information will be presented on epilepsy and DTI, and approaches for DTI use, as mostly seen in human medicine, will be explored in the context of veterinary medicine.

### 1.1 Epilepsy

A seizure is a transient and abnormal increase in synchronization between neurons. Seizures are epileptic when two or more episodes occur at least 24-h apart (28). Seizure types can be described as generalized, focal, or unknown based on their cortical origin. The term ‘generalized seizure’ refers to synchronized neuronal activity originating in networks that engage both hemispheres. A ‘focal seizure’ refers to seizure activity originating in one hemisphere. Focal seizures can evolve to bilateral tonic–clonic seizures when activity progresses to generalized activity within a seizure episode. Lastly, unknown seizure type occurs when the hemispheric location of onset of seizure activity is unknown (28, 29). Epilepsy is an enduring predisposition to the occurrence of epileptic seizures. In people, it is classified by seizure type, with implications as to comorbidities, therapeutic recommendations, and outcomes (30, 31).

The focus of the present review is on canine IE; in other words, epilepsy with a known or suspected genetic influence or an unknown cause (6, 28). Similarly, in human medicine, when genetics are known to play a role, IE is referred to as genetic epilepsy. Human medicine has a wider range of defined epilepsy types and syndromes within IE (30, 31). Veterinary medicine is working towards the development of syndrome specificity within IE (32, 33).

There are three tiers of confidence for diagnosing IE in dogs. Tier I is used when two or more epileptic seizures occur at least 24 h apart, signs of epilepsy appear between approximately 6 months and 6 years of age, neurological examination during interictal periods is normal, and baseline blood analysis and urinalysis are unremarkable. Tier II confidence is used when Tier I criteria is fulfilled and no underlying causes are detected using additional blood tests, urinalysis, bile acids tests, cerebrospinal fluid analysis, and magnetic resonance imaging (MRI) of the brain (6). Although standard MRI sequences for the epileptic canine brain are expected to be unremarkable, a few reports exist of hippocampal atrophy or other qualitative or quantitative abnormalities on routine MRI sequences (34, 35). However, it is important to differentiate between post-ictal and interictal abnormalities (36). Cases of post-ictal changes, mainly localized to the piriform lobe, temporal lobe, cingulate gyrus, and hippocampus, have shown a marked reduction to full resolution on follow-up MRIs (36–38). Interictal parenchymal abnormalities are more common among older dogs with IE in regions such as the frontal lobe, piriform lobe, and occipital lobe (39). Tier III diagnostic criteria include all criteria from Tier II plus evidence of seizure activity using electroencephalography (EEG). While a standard protocol for electrode placement exists in human medicine, veterinary medicine is still working towards verifying electrode placement for adequate coverage of the superficial cortical layer in dogs (6, 40–44). Even so, EEG data of an ictal event or interictal epileptogenic patterns still offers the highest level of confidence in the diagnosis of IE (6). This has its challenges as there is a lower likelihood of capturing an ictal or interictal electrographic event in dogs with less frequent seizures (41). Having a microstructural marker that is not reliant on real-time events during neuroimaging would provide another method for obtaining diagnostic confirmation during interictal periods.

#### 1.1.1 Drug resistant epilepsy

While most individuals with IE are successfully treated using ASMs, approximately 25 to 35% of people and dogs have drug resistant epilepsy (DRE) (45–50). The International League Against Epilepsy defines DRE as the failure to reach seizure freedom using two or more ASMs (51). In clinical trials for veterinary medicine, DRE is often referred to as the inability to reduce seizure frequency by at least 50% using two or more ASMs (45–48, 52, 53). The more ASMs being taken, the higher the probability of experiencing unpleasant adverse effects (54). While people might verbally report adverse effects, veterinarians must rely on caregiver observations, behavioral signs, and physiological signs to detect side effects in dogs (6, 53). Therefore, detecting adverse effects is more difficult in dogs than in people. One example of a polytherapy adverse effect that may go unnoticed in dogs versus people is cognitive dysfunction (8). While alternatives to polytherapy are being explored, the overall task of treating and controlling DRE remains a big challenge for both species (3, 53–55).

### 1.2 Diffusion tensor imaging (DTI)

**Figure 1:**
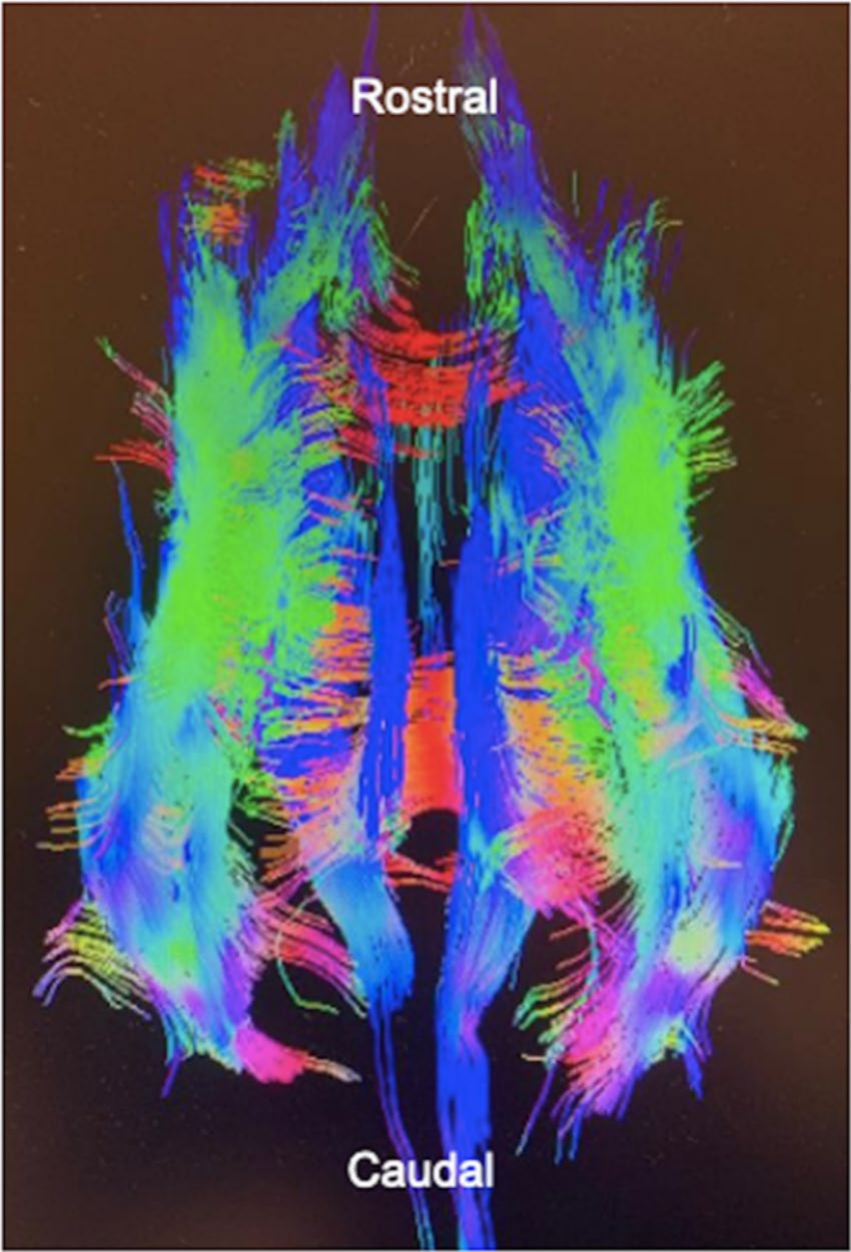
A diffusion tensor image of a dog’s brain. Tractography is used to predict fiber tract direction which is represented by color. Created with General Electric AW VolumeShare 7.

Diffusion tensor imaging is an MRI sequence based on an algorithmic model. It incorporates data from diffusion weighted images (DWI) taken in multiple planes to form a three-dimensional image. Diffusivity of water molecules is used to highlight structural connectivity patterns of large white matter tracts (Figure 1) (11). Diffusion patterns in the brain are dependent on the density, permeability, and the direction of axons, large molecules, and microstructures.

**Figure 2:**
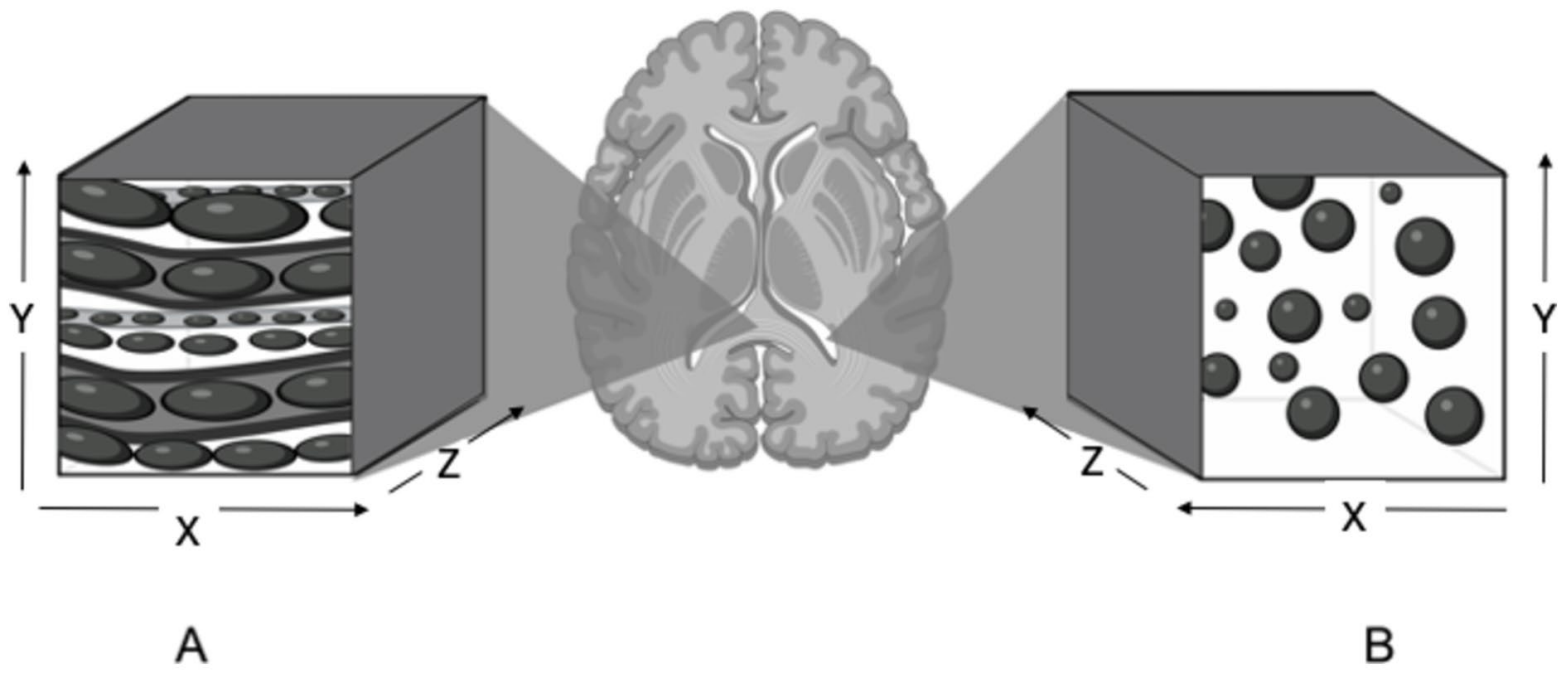
Illustration of anisotropic and isotropic diffusion in selected regions of interest in the brain. **(A)** Anisotropic diffusion observed in the corpus callosum. Water molecules are restricted by dense axons and diffuse in a parallel and elongated fashion. **(B)** Isotropic diffusion observed in the lateral ventricle. Water molecules move in an outwards and spherical manner in the absence of structural barriers. Created with BioRender.com.

Diffusion can be referred to as isotropic or anisotropic (Figure 2). Isotropic diffusion is the movement of molecules in an outwards and spherical direction in the absence of structural barriers. The mean magnitude of isotropic diffusion is measured using an apparent diffusion coefficient (ADC). The mean diffusivity (MD) is calculated using ADC values in three orthogonal or more directions. Higher values for these indices are related to increased extracellular space, less structural organization, and fewer axons (11, 21). Anisotropic diffusion refers to the tracking of water molecules within and along densely packed axonal tracts, i.e., the parallel movement of water molecules. Diffusion tensor imaging uses the magnitudes of multiple ADC values in three or more planes to measure the proportion of fractional anisotropy (FA) within a region of interest (ROI). A higher FA value indicates structural organization and a dense region of parallel axons. Dense regions of parallel axons make up large white matter tracts (11, 21, 56).

Diffusion indices can be measured in segments of tracts, tracts, and whole brain white matter. Connectivity indices are used to structurally or functionally analyze networks. Quantitative information on network structure and function can be extracted from DTI using mathematical approaches such as graph theoretical analysis and independent component analysis (14, 19, 57, 58). Indices calculated from graph theoretical analysis highlight the strength, length, and type of connections being made between regions of the brain (19, 58). Independent component analysis allows for the breakdown and buildup of spatiotemporal activity of each voxel (14, 57). Both are useful for understanding brain connectivity and differentiating between normal and abnormal structure or function (14, 19, 57, 58).

Regardless of whether segments, tracts, whole brain white matter, or networks are used as ROIs, each method has benefits and pitfalls (59–61). Segments of white matter tracts allow the selection of regions with lower levels of crossover but are limited by a smaller number of voxels. Conversely, thin slices on image acquisition with zero spacing between slices could be used to increase the number of voxels being selected. Crossover refers to fiber tracts going in different directions within the same voxel and is inevitable when analyzing tracts and whole brain white matter (60, 61). Qualitatively, the overlap from crossing fibers makes it hard to visually differentiate tracts using FA maps, color orientation maps, and tractography. As FA is a vector, and the magnitude of its value is dependent on direction, when tracts cross because they are going in different directions, FA values are cancelled out (59–61).

Tracts and whole brain white matter can also be analyzed using tract based spatial statistics (TBSS); a reliable method for calculating accurate FA values. This method requires distorting, or transforming, each participant’s brain images to fit a standard anatomical reference; because of the varying skull morphology in dogs, this task can be challenging and labor intensive but is possible for mesocephalic dogs using a brain atlas space (62). Network measures account for the broad impact of epilepsy but may miss subtle nuances. Furthermore, functional connectivity measures are limited to resting state networks, such as the default mode network, in dogs as they almost always require anesthesia for MRI scans. Resting state networks refer to baseline connections between different functional regions of the brain. These networks highlight brain activity that does not require awareness to elicit measurable changes (63–66). However, it is possible that resting state networks are being functionally altered by anesthesia (64–66).

## 2 Diffusion tensor imaging in people with epilepsy

People with various types of epilepsy show a trend of decreased anisotropic diffusivity and/or an increase in isotropic diffusivity. Examining patients with generalized genetic epilepsy (GGE), decreased FA and increased MD was found in the corpus callosum, corticospinal tract, superior and inferior longitudinal fasciculus, and supplementary motor areas (12). Similarly, low FA and high perpendicular diffusivity (an additional measurement of isotropic diffusivity) were seen in the posterior corpus callosum, external capsule, internal capsule, and anterior corpus callosum of patients with temporal lobe epilepsy (TLE) (7). Temporal lobe epilepsy is a common form of focal epilepsy in people where activity originates in the temporal lobe (67). Results from these studies imply that the white matter regions in those with GGE and TLE have more extracellular space and less dense axonal tracts. These studies, among others, highlight the widespread impact that GGE and TLE can have on the brain. As well, they feed into the growing consensus that epilepsy influences or involves structures outside of epileptogenic zones, the predicted origin of seizure activity (7, 12, 17, 68–71).

While white matter structures outside of epileptogenic zones show reduced FA in people with epilepsy, the closer to the epileptogenic zone, the larger the FA reduction. This means there tends to be lower FA in the hemisphere containing the epileptogenic zone (7, 15, 18). Similarly, an ipsilateral reduction in FA is seen in the hippocampal-thalamic pathway of individuals with TLE who experience generalized seizures when compared to controls (10). Another study found an increase in the ADC of the hippocampus located in the hemisphere that initiates seizure activity in those with IE (72). This suggests asymmetry between hemispheres is detectable and should be considered a potential variable in DTI studies of IE. It also suggests that the more brain regions are exposed to seizure activity the more effects are seen, meaning seizure frequency should be considered also as a variable that influences brain microstructure.

People with TLE and hippocampal sclerosis (TLE-HE), TLE, and GGE, show a reduction in FA in multiple white matter regions with the lowest FA values in those with TLE-HE (71). Hippocampal sclerosis refers to the loss of cells within the hippocampus (70). Age of onset and duration with epilepsy is negatively correlated with FA in people with TLE but to a greater degree in those with TLE-HE (7, 71). This implies that the age of onset may influence the extent to which epilepsy changes the structural integrity of regions of white matter that are still developing. The progressive decrease in FA seen in those with TLE, in combination with the greater extent of damage and higher correlation between duration and FA reduction in patients with TLE-HE, could imply quantitative changes are detectable prior to visual detection of structural abnormalities in those with IE.

Functional and structural connectivity abnormalities of neuro-networks are detected in people with IE (14, 19, 20). Functionally, a decrease in connectivity in the default mode network is reported in people with ASM-resistant generalized IE when compared to healthy controls (14). Independent component analysis identified functional connectivity changes in people with juvenile myoclonic epilepsy (JME). There was enhanced connectivity between the prefrontal cortex and the motor cortex, as well as between the supplemental motor areas and lateral and caudal regions of the brain. Supplemental motor areas also showed a decrease in connectivity with rostral regions of the brain (20). Juvenile myoclonic epilepsy is a prominent IE syndrome in humans that shares parallels with JME in Rhodesian Ridgeback dogs (20, 32, 33).

In terms of structural connectivity, there are differences in whole brain networks between people who are good responders versus poor responders to ASMs. Newly diagnosed people with focal epilepsy, naïve to ASMs, had DTI scans prior to determining if they were good-responders to ASMs (IE+) or poor-responders to ASMs (IE-). To meet the criteria for IE+, participants needed to become seizure free for 6 months or more. The mean assortative coefficient, calculated using graph theoretical analysis, was positive for IE+ and negative for IE-. In other words, good responders had more connections between similar brain regions and poor responders had more connections between dissimilar brain regions (19). This provides evidence of microstructural differences in white matter between people who are responsive versus resistant to ASMs.

This body of literature exemplifies a wide range of abnormalities that can be detected using DTI in various presentations of epilepsy (7–21, 68–73). Given the similarities between epilepsy in people and dogs, research from human medicine provides a methodological starting point for what should be explored in veterinary medicine. Conversely, the translational value of canine studies means that they can serve as a valuable source of insights for human medicine (22–26).

## 3 Translation of DTI literature in people to dogs

People and dogs have similar neuroanatomy and, further, share many aspects of epilepsy such that each could be considered a model for the other (22–26). For example, because the proportion of those with DRE is similar in people and dogs, it is possible that the mechanisms responsible for drug resistance are similar between species (3, 9, 19, 53, 63). More research would be needed to factually support this statement. As DTI is a relatively new technique used to analyze connectivity within the brain, and most published research on DTI and epilepsy has been studied in people, trends from human medicine provide a framework for designing DTI studies for dogs with IE (7, 11, 14, 17, 19, 74).

Conversely, there may also be limits to the transferability of data between human medicine and veterinary medicine. For example, there is a significant difference between the connectivity of the anterior cingulate cortex and posterior cingulate cortex when comparing neurotypical people and dogs. More specifically, the dogs had lower anisotropic diffusion between their anterior and posterior cingulate cortex (57). Epilepsy in people has been correlated with lower anisotropic diffusion in multiple white matter structures; thus, using human medical literature to interpret canine data may result in inaccurate conclusions (14, 57, 63). Therefore, overall trends from human medicine should be considered but actual FA values of ROIs are not reliable measures to compare between the species.

### 3.1 Selecting regions of interest for dogs with idiopathic epilepsy (IE)

Epilepsy affects the microstructure and function of white matter tracts and influences the connectivity patterns within and between neuronal networks. Based on this, it is reasonable to use either segments of tracts, tracts, whole brain white matter, or networks as ROIs (7–21, 68–72).

Segments of white matter tracts investigated in people with IE include the corpus callosum, cingulum, external capsule, internal capsule, mammillothalamic tract and hippocampus (7, 12, 17, 69–73, 75). Depending on breed, MRI quality, and software constraints, the external capsule and mammillothalamic tract may be too small for voxel selection in dogs. However, the corpus callosum, cingulum, internal capsule and hippocampus are prominent structures that could be used as ROIs for dogs (27, 56, 76).

Whole tracts studied in people with IE that could be used as ROIs in dogs include the corticospinal tracts, superior longitudinal fasciculi, interior longitudinal fasciculi, and hippocampal-thalamic pathway (10, 12, 17, 74). In dogs, the corticospinal tract is hard to differentiate from the corticobulbar and corticopontine tracts meaning these may need to be analysed together (56).

The default mode network and overall brain networks could be used as ROIs when studying functional and structural connectivity (14, 17, 19). While other networks may be involved in epilepsy, such as the thalamocortical network or basal ganglia, these regions would require functional MRI techniques and unanesthetized dogs, making for a more complex experimental protocol and timeline (57, 63, 77).

## 4 Diffusion tensor imaging in dogs

As DTI is a relatively new technique, only a few studies have demonstrated the feasibility of its use in a veterinary context (27, 56, 63, 78–81). Extensive mapping of separate white matter tracts in healthy dogs using DTI has been verified with cadaveric dissections and anatomy textbooks, creating atlases. Such atlases could be used to compare tracts between healthy dogs and dogs with IE (56). Other atlases focused on parcellation of subcortical and cortical grey matter regions. While individual tracts and structures of white matter were not parcellated, segmentation of whole white matter was defined (62, 79, 82–86). Grey matter abnormalities are less of a focus in DTI but a significant increase in ADC in the piriform lobes of dogs with IE has been reported (87). Meaning, parcellation of these grey matter regions could be useful for DTI research. Furthermore, identifying both grey and white matter abnormalities comes into play in regions like the hippocampus, a portion of the piriform lobe with a mixed composition of grey and white matter. In terms of whole brain white matter, at least one of these atlases are being used to standardize participants’ brains for TBSS (62).

An increase in isotropic diffusivity and decrease in anisotropic diffusivity in the hippocampus of people with TLE without HE provides evidence that quantitative imaging may precede visually recognizable structural abnormalities (71, 72). Similarly, in a study on dogs with IE, there was an increase in hippocampal atrophy, from 12 to 36%, when calculated visually versus using the hippocampal asymmetry ratio (35). Hippocampal atrophy is sometimes present on MRI scans in relation to IE which is a discrepancy with IVETF tier II criteria stating that there are no structural brain abnormalities in dogs with IE (6, 34). The hippocampal asymmetry ratio is dependent on volumetric measures. Therefore, other quantitative measures, such as diffusion indices, may also be sensitive to microstructural abnormalities in the brains of dogs with IE.

Asymmetry between hemispheres may be a feature to explore in dogs with IE. Notably, a certain amount of FA asymmetry is to be expected in some regions based on what is reported in people. For example, the left corticospinal tract tends to have higher FA than the right corticospinal tract in healthy people (74). The corticospinal tract has been reported as an ROI for FA reduction in people with IE (12). Therefore, a thorough understanding of asymmetry in healthy individuals would need to be investigated prior to making any conclusions related to IE in dogs.

The other route for studying microstructural and functional abnormalities in dogs with IE is to look at DTI formulated probabilistic representations of neuro-networks (14, 19, 57, 58). Graph theory analysis could be applied to DTIs in dogs with IE, using methodological frameworks published in human medicine, to enable the investigation of structural abnormalities of connectivity (19). Functionally, the anterior region of the default mode network in dogs with IE shows an increase in connectivity (57). In this example, results differed from a similar study in people with general IE (14). While these results are contradictory, it was theorized that there may be a compensatory increase in connectivity prior to degradation that occurs over the course of the disease (57). The same concept was discussed in a study that found an increase in FA in the frontal lobe of newly diagnosed and treatment naïve children with generalized IE (16).

### 4.1 Diffusion tensor imaging in dogs with IE

Only one article has looked at DTI in dogs with IE. Notably, the authors selected a couple ROIs that have been found to have reduced FA in people with epilepsy, namely, the corpus callosum and cingulate white matter. They reported a significant decrease in FA in the cingulate white matter of dogs with IE. Tract based spatial statistics of this cingulate white matter did not show the same significant decrease in FA which may be because TBSS does not highlight subtle microstructural differences (27). These findings could be expanded by using a larger population and additional ROIs.

## 5 Additional variables to consider for DTI research in dogs with IE

Breed and age affect brain microstructure in canines and sex and age affect brain microstructure in humans (7, 17, 81, 88–92). Morphological variability exists between dog breeds, where specific behaviors correlate with structural differences in the brain (89). Moreover, human studies have shown differences in brain diffusion and connectivity between sexes. For example, one study reports that males have higher FA in multiple white matter regions when compared to females (91). Another large study found males have more intrahemispheric connections while females have more interhemispheric connections (90). In terms of age, neuronal degradation in people, which is reflected by a decrease in FA, generally starts in the anterior region of the brain and continues posteriorly (92). Therefore, age at scan may negatively correlate with FA in certain regions of the brain (7, 81, 92). In relation to dogs, Barry et al. (81) studied the influence of age on white matter FA in a sample of 29 healthy mesaticephalic dogs. The dogs were divided into two age categories: the young group included dogs under the age of 7, and the old group included dogs aged 7 or older. They found a significant decrease in FA in multiple white matter regions, including the corpus callosum, in the old group when compared to the young group (81). Note, a decrease in FA in relation to age is not noticeable before the age of 30 in people (88); meaning, a pattern may not be evident in a sample of mainly young dogs. Overall, breed, sex, and age should be considered when determining the relationship between white matter microstructure and IE in dogs.

Volume is another factor to consider when studying IE in dogs. While volume is not the focus of this review, volumetric data is simultaneously provided when selecting ROIs from DTIs for diffusion analyzes (93). Nuyts et al. (17) conducted a meta-analysis on structural abnormalities associated with generalized IE in people. They reported a statistically significant reduction in volume in the supplemental motor area, insula, thalamus, putamen, caudate, hippocampus, anterior cingulate cortex, and left pallidum. Also, the medial frontal gyrus was larger in the right hemisphere of those with generalized IE compared to controls (17). Furthermore, Milne (94) reported that dogs with IE had a widespread reduction in cerebral cortical volume when compared to healthy controls (94). While volume of structures can vary between individuals, overall volumetric patterns and ratios are worth investigating as all the information needed would already be obtained (17, 89).

## 6 Discussion

The question of whether DTI is a viable resource for detecting microstructural white matter abnormalities in the brains of dogs with IE needs further investigation. Comparisons using DTI indices have yet to be conducted between dogs with IE+ and IE−. A prospective case–control cohort study design would be optimal for determining whether there are microstructural differences between healthy dogs, IE+, and IE−. Healthy dogs and dogs with newly diagnosed IE, naïve to ASM treatment, could receive an initial scan and a follow up scan 12 months later. During these 12 months, dogs with IE would be treated with ASMs and categorized as ASM responsive or resistant following IVETF definitions. Diffusion indices and structural connectivity measures could be blindly analyzed. Comparisons could be made (1) between neurotypical dogs and dogs with IE to control for confounds; (2) between the initial scans of dogs that are resistant versus responsive to ASMs to determine the presence of biomarkers; (3) as well as between and within arms over time to account for additional confounds and compare progressional changes in diffusion and connectivity of the brain.

The first comparison controls confounds such as breed, sex, and age. The second comparison would be useful for determining whether microstructures detected by DTI could be used as biomarkers to help predict ASM resistance; in turn, adding more specification to diagnoses and allowing for more informed treatment planning. Structural connectivity has been compared between people who are good versus poor responders to ASMs (12). Assortative connectivity was associated with good responders and disassortative connectivity was associated with poor responders. With the similarities between human and canine epilepsy, these results may translate to dogs. The range of structural networks that have been explored using DTI in humans is vast, whereas the most practical functional networks to investigate in dogs with IE are resting state networks (14, 19, 57, 63–66). In addition to network measures, anisotropic diffusivity in segmentations of tracts, tracts, or whole brain white matter could be investigated. Segments of tracts previously implicated in human IE that are translatable to dogs include the corpus callosum, cingulate gyrus, internal capsule, and hippocampus. Furthermore, tracts include the corticospinal, corticobulbar, and corticopontine tracts, the superior and inferior fasciculi, and hippocampal-thalamic pathway (7, 10, 12, 17, 69–71, 73–75). If diffusion indices are investigated, Figley et al. (95) emphasizes the importance of using multiple measures to validate FA findings. This is because the ratio of neurons going in the principal direction is being calculated, not overall density of fibers within a voxel. For instance, FA could be higher in an area with fewer parallel fibers than in an area with densely packed fibers going in multiple directions. Also, damaged white matter may not result in a change in FA if fibers in all directions are equally damaged; this is because the ratio between the fibers going in different directions would remain the same (95). Furthermore, histological findings suggest that ADC, an isotropic diffusion index, is a better measurement of myelination than FA, as FA measures myelination among other microstructural constraints of water diffusion. Different diffusion indices offer some overlap and some variation in information (95–97). Therefore, more detailed information can be obtained using multiple diffusion indices when studying IE.

Thirdly, asymmetry comparisons over time between hemispheres should be made as differences have been exemplified in people and dogs with IE (7, 15, 18, 35, 71, 73). More importantly, it would provide information about the progression of changes in ASM responsiveness versus resistance in patients. As well, act as a within control measure to gain pilot data for future investigations on additional factors that are potentially influencing microstructural change such as number of ASMs being taken, ASM type, seizure frequency, and seizure type.

## 7 Conclusion

Research is building on evidence of microstructural abnormalities in dogs with IE from DWI, volumetric studies, and one DTI study (27, 35, 87, 94). Functional abnormalities in the default mode network of dogs with IE have been identified (57). Further investigations will be needed into anisotropic diffusivity and structural connectivity measure in dogs with IE. While DTI is a useful tool to investigate brain abnormalities related to epilepsy in people, findings from human medicine are not always transferable to veterinary medicine (7, 11, 12, 14, 17, 19, 57, 73). On this front, human medicine is ahead of veterinary medicine and provides a valuable framework to guide veterinary research. In turn, veterinary medicine may provide valuable information for human medicine (22–26). There are many types of ROIs and measures that could be used, and each one comes with benefits and limitations. As well, confounds such as breed, age, sex, asymmetry, ASM specifications and seizure or epilepsy type specifications would need to be considered. In tandem with other diagnostic techniques, discoveries using DTI could lead to more specific diagnoses and targeted treatments for dogs with IE (12, 17, 19, 32, 33, 57, 95, 97).
